# Minodronate treatment improves low bone mass and reduces progressive thoracic scoliosis in a mouse model of adolescent idiopathic scoliosis

**DOI:** 10.1371/journal.pone.0202165

**Published:** 2018-08-23

**Authors:** Hironori Tanabe, Yoichi Aota, Yasuteru Yamaguchi, Kanichiro Kaneko, Sousuke Imai, Masaki Takahashi, Masataka Taguri, Tomoyuki Saito

**Affiliations:** 1 Department of Orthopedic Surgery, Yokohama City University, Yokohama, Japan; 2 Department of Spine & Spinal Cord, Yokohama Brain & Spine Center, Yokohama, Japan; 3 Yokohama City University Center for Novel and Exploratory Clinical Trials, Yokohama City University, Yokohama, Japan; 4 Department of Biostatistics, Yokohama City University, Yokohama, Japan; Garvan Institute of Medical Research, AUSTRALIA

## Abstract

Recent studies have shown an association between osteopenia and adolescent idiopathic scoliosis (AIS) and implied that osteopenia plays a causative role in AIS development. This study aimed to determine if minodronate (MIN) treatment could prevent curve progression by increasing bone mass in a thoracic restraint (TR) mouse model, which develops causes the development of thoracic scoliosis similar to human AIS. A total of 100 young female C57BL6J mice were divided into four groups: (1) control with vehicle (CON/VEH; n = 20), (2) control with MIN (CON/MIN; n = 20), (3) TR with vehicle (TR/VEH; n = 30), or (4) TR with MIN (TR/MIN; n = 30). MIN (0.01 mg/kg/week) and vehicle were administered intraperitoneally to their respective groups. TR was performed at age 4 weeks, and the mice were sacrificed at age 9 weeks. Body weights, spine radiographs, femoral bone mineral density (BMD), serum bone marker levels, and histomorphometry of the cancellous bone of the thoracic vertebrae were analyzed. TR significantly reduced weight gain in the TR/VEH group relative to the CON/VEH group. TR also induced osteoporosis with accelerated bone resorption, as indicated by decreases in femoral BMDs and thoracic cancellous bone volume and increases in serum bone resorption marker levels and histomorphometric resorption parameters in the TR/VEH group. MIN partially improved body weight gain and improved poor bone structure relative to the TR/VEH group by suppressing high bone resorption in the TR/MIN mice. MIN significantly reduced the curve magnitudes, as indicated by a 43% lower curve magnitude in the TR/MIN mice than in the TR/VEH mice (17.9 ± 8.9° vs. 31.5 ± 13.1°; p< 0.001). The administration of MIN increased bone mass and reduced the severity of scoliosis in the TR mice. MIN was suggested as a possible inhibitor of scoliosis development.

## Introduction

Adolescent idiopathic scoliosis (AIS) is a complex three-dimensional structural deformity of the spine. The prevalence of AIS ranges from 2% to 4% [1–3], especially during pre-pubertal and pubertal growth, when bone acquisition is highest. Poor bone mineral density (BMD) has been associated with AIS in 27–38% of cases [4–6], and osteopenia may be a primary contributing factor to the spinal deformity of AIS, rather than a secondary outcome [4]. Low BMD has been reported to be a significant prognostic factor of curve progression in AIS [7,8]. Although the cause of low bone mass in AIS has remained ill-defined, a recent study has indicated that higher rates of bone resorption may contribute to low bone mass in patients with AIS [9].

In patients with AIS exhibiting severe progressive curvatures, the spinal deformity causes a disturbed self-image and potential health problems associated with cardiopulmonary function and back pain [10]. About 10% of patients with AIS have significant spinal deformities that require treatment [3,11]. Only bracing and surgery have been accepted as treatments for AIS, with the implication that the reconstruction of spine balance and improvement in bone strength would help prevent curve progression [12]. However, many patients with AIS continue to experience residual problems because of poor treatment compliance [13] and surgical complications [14]. Therefore, investigating new therapeutic approaches is essential.

Bisphosphonates are widely used for patients with osteoporosis because of their inhibition of osteoclastic bone resorption and the consequent increase in BMD, and their bone strengthening effects [15–17]. Minodronate, a third-generation bisphosphonate with an imidazopyridine ring side chain, is the strongest inhibitor of bone resorption and has the lowest required concentration for bone resorption inhibition among bisphosphonates [18–20]. Spinal deformities are also common in patients with osteogenesis imperfecta (OI), a heritable disorder that causes bone fragility. Bisphosphonate therapy improves vertebral bone quality and decreases the curve progression rate in patients with OI type-III [21,22]. Anti-osteoporosis treatment may provide an internal force to the trunk during the growth phase, which can prevent curve progression in pediatric patients having spinal deformity. Hence, we hypothesized that bisphosphonate treatment could also help prevent curve progression in AIS. Thoracic restraint (TR) induced underdevelopment of the anteroposterior dimension of the rib cage, provoked an imbalanced load on the vertebral column, and consistently produced thoracic scoliosis in mice with anatomic characteristics similar to those of human patients with idiopathic scoliosis [23]. In this study, we examined whether the progression of scoliotic curves in TR mice could be inhibited by the administration of minodronate.

## Materials and methods

### Overview of study design

One hundred female young mice (C57BL/6J 4 weeks of age; Japan SLC, Shizuoka, Japan) were either subjected to TR or left as untreated controls (CON), and injected once weekly with either minodronate (MIN, 0.01 mg/kg/week, intraperitoneally; Tokyo Chemical Industry Ltd., Tokyo, Japan) or a vehicle (VEH). Thus, the mice were assigned to one of the four groups: CON/VEH (n = 20), CON/MIN (n = 20), TR/VEH (n = 30), or TR/MIN (n = 30) by body weight to minimize differences between the groups at baseline. The mice were maintained under 12-hour light/dark periods at a constant room temperature (23° ± 2°C), with food and water provided ad libitum. All animal experiments were conducted in accordance with a review-board-approved protocol at Yokohama City University School of Medicine (F-A-15-021).

### Thoracic restraint procedure

To limit the anteroposterior rib cage development in mice, plastic device was applied to the chest of each mouse at 4 weeks of age, under isoflurane anesthesia, as previously described [23]. To avoid detachment of the TR equipment after application, the restraints were fixed to the body of the mice with 5–0 nylon sutures, from both the dorsal and ventral sides of the body. After the restraint application, the mice were returned to their normal housing conditions.

### Sample preparation

At the age of 9 weeks, the mice were randomly selected from each group (n = 6 for CON/VEH, CON/MIN and TR/VEH, and n = 9 for TR/MIN), and blood samples were obtained by an intracardiac puncture after administration of isoflurane anesthesia. The blood sample was kept at room temperature (23° ± 2°C) for 1 hour and then placed on ice. The samples were centrifuged at 3000 rpm for 10 min at 4°C. Serum sample was collected and stored at −20°C until the bone turnover marker analysis. The remaining mice were sacrificed via CO_2_ inhalation. The thoracic vertebral column and right femur that were randomly selected from the mice of each group (n = 6) were then dissected and preserved in 70% ethanol at 4°C for analyses of femoral BMD, and bone histomorphometry of the thoracic vertebrae. Randomization of the samples for each experiment was performed using a computer-generated random allocation sequence by simple randomization.

### Body weight

The mice were weighed from 4 to 9 weeks of age (n = 20 for CON/VEH and CON/MIN, and n = 30 for TR/VEH and TR/MIN).

### Measurement of Cobb angle

At 9 weeks of age, a posteroanterior radiograph of the vertebral column of each mouse was obtained under isoflurane anesthesia, using an in vivo micro-CT system (R_mCT 2; Rigaku, Tokyo, Japan) (n = 20 for CON/VEH and CON/MIN, and n = 30 for TR/VEH and TR/MIN). The TR was removed before imaging, and the mice were positioned in a prone position. Radiographs were obtained thrice, and the image obtained in the most symmetrical position with regard to the shoulder and hip joints was used. The severity of spinal deformity was evaluated using R_mCT 2 image analysis software program (Rigaku). Scoliosis was defined as a Cobb angle [23] greater than 10°, and severe scoliosis was defined as a Cobb angle greater than 40°. All measurements were obtained by three investigators, using a double-blind technique. The deformities were evaluated for the incidence of scoliosis and the severity of the curves between the TR/VEH and the TR/MIN groups.

### Measurement of femoral BMD

The femoral BMD of the mice at the age of 9 weeks (n = 6 for each group) was measured by using dual-energy X-ray absorptiometry (DXA) with the DCS-600EX-R system (Hitachi Aloka Medical, Ltd., Tokyo, Japan).

### Bone turnover markers

All serum measurements from the mice at the age of 9 weeks (n = 6 for CON/VEH, CON/MIN and TR/VEH, and n = 9 for TR/MIN) were performed as indicated by the manufacturer. The bone formation marker, osteocalcin (OCN), measurement was performed using the Mouse enzyme-linked immunosorbent assay kit (Cloud-Clone Co., Houston, USA). The bone resorption marker, tartrate-resistant acid phosphatase 5b (TRACP 5b), measurement was performed using the Mouse TRAP enzyme-linked immunosorbent assay kit (Immunodiagnostic Systems, Boldon, UK).

### Bone histomorphometry of the thoracic vertebrae

To examine bone formation rates, tetracycline hydrochloride (20 mg/kg; Sigma-Aldrich, St. Louis, USA) and calcein (20 mg/kg; Dojindo Laboratories, Kumamoto, Japan) were subcutaneously injected 4 days and 2 days before death, respectively, to double-label the bones. After the removal of soft tissue, the whole thoracic vertebrae from each group (n = 6) were subjected to Villanueva bone staining for 7 days without a decalcifying treatment [24], dehydrated with increasing concentrations of ethanol, and embedded in methyl methacrylate (Wako Pure Chemical Industries, Osaka, Japan). Sagittal sections of the vertebral body (5-μm thick) were prepared using a microtome (LIECA, Wetzlar, Germany). Fifteen fields of the secondary cancellous bone per vertebra on the T6, T7, and T8 were measured, and the means of the measured values were used for data analysis. A single experienced investigator measured the parameters of each specimen. The nomenclature and units used were in accordance with the ASBMR Histomorphometry Nomenclature Committee [25].

The parameters measured for bone structures were total bone volume per tissue volume (BV/TV, %), trabecular thickness (Tb.Th; μm), trabecular number (Tb.N, /mm), and trabecular separation (Tb.Sp, μm). The parameters obtained for bone formation were osteoid surface per bone surface (OS/BS, %), osteoblast surface per bone surface (Ob.S/BS, %), and osteoid thickness (O.Th, μm). The parameters measured for bone resorption were eroded surface per bone surface (ES/BS, %), osteoclast number per bone surface (N.Oc/BS), and osteoclast surface per bone surface (Oc.S/BS, %). The parameters obtained for bone dynamics were mineralizing surface per bone surface (MS/BS, %), mineral apposition rate (MAR, μm/day), and bone formation rate per bone surface (BFR/BS, μm^3^/μm^2^/year).

### Correlations between Cobb angle and contributing factors

To determine the contributing factors to scoliotic curves in this model, correlations among the Cobb angle, histomorphometric parameters, and femoral BMDs were analyzed in the minodronate-untreated TR mice (TR/VEH group, n = 6).

### Statistical analysis

All data are expressed as mean ± SD. To compare means among the groups, the two-way ANOVA was used, and post-hoc pairwise comparisons were conducted using the Dunnett's test. Differences in body weight among the groups were determined using a repeated-measures ANCOVA with the 4-week body weight as the covariate. Proportional differences were analyzed using the Fisher’s exact test for independence. The linear associations between the Cobb angle, histomorphometric parameters, and femoral BMDs in the TR/VEH group were assessed using the Pearson correlation coefficient. The calculations were made using the SPSS 15.0 for Windows. A p-value of < 0.05 was considered significant for all statistical analyses.

## Results

### Growth of TR mice and the effect of minodronate on body mass

**Fig 1:**
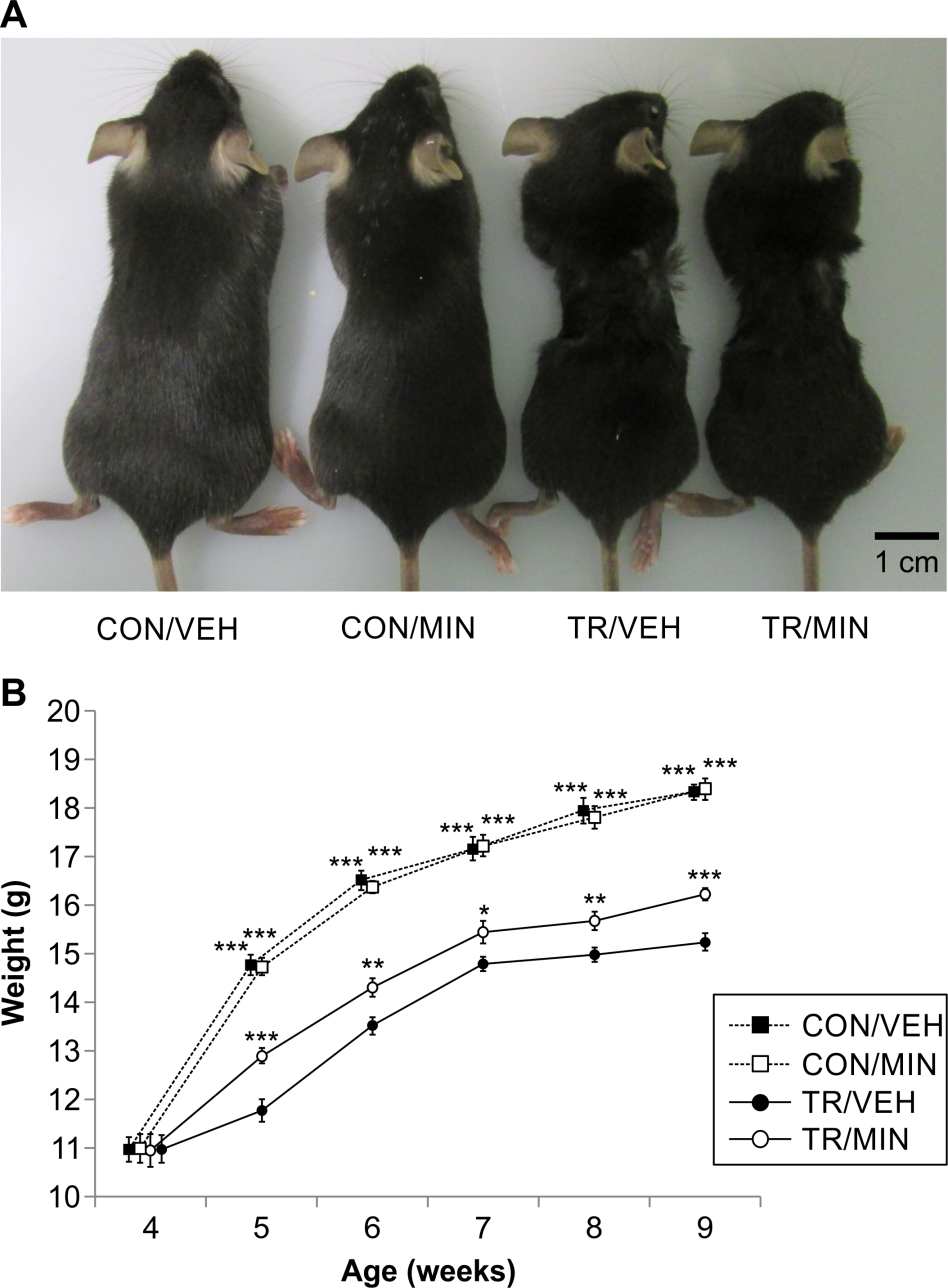
Thoracic restraint (TR) impairs the body growth of mice, and minodronate treatment inhibits TR-induced skeletal deterioration in mice. (A) Representative photographs of the CON/VEH, CON/MIN, TR/VEH, and TR/MIN mice at 9 weeks of age. TR treated mice appear to be emaciated. Scale Bar, 1 cm. (B) Total body weights of the CON/VEH, CON/MIN, TR/VEH, and TR/MIN mice at age of 4–9 weeks. Data are presented as mean ± SD (n = 20 for CON/VEH and CON/MIN, and n = 30 for TR/VEH and TR/MIN; *p< 0.05, **p< 0.01, and ***p< 0.001, significantly different from the TR/VEH group as reference (Dunnett’s test)).

The representative appearance of the CON/VEH, CON/MIN, TR/VEH, and TR/MIN mice is presented in Fig 1A. The CON/VEH and CON/MIN mice exhibited normal body growth, whereas the TR/VEH and TR/MIN mice appeared to be emaciated at 9 weeks of age. Limb paralysis and abnormal ambulation were not found in any mice. Body weight changes between 4 and 9 weeks of age for the mice of each group are presented in Fig 1B. Similar body weight patterns were observed in the CON/VEH and CON/MIN group. Meanwhile, the values of body weight were significantly lower in the TR/VEH group than in the CON/VEH group at 5 to 9 weeks (p< 0.001, at each week), suggesting TR impaired weight gain. Minodronate showed a positive effect on body weight in the TR/MIN mice, which weighed more than the TR/VEH mice at 5 to 9 weeks (p< 0.05 at 7 weeks, p< 0.01 at 6 and 8 weeks, p< 0.001 at 5 and 9 weeks), suggesting that minodronate treatment partially counteracted TR-induced reduction in body weight gain.

### Effect of minodronate on Cobb angle

**Fig 2:**
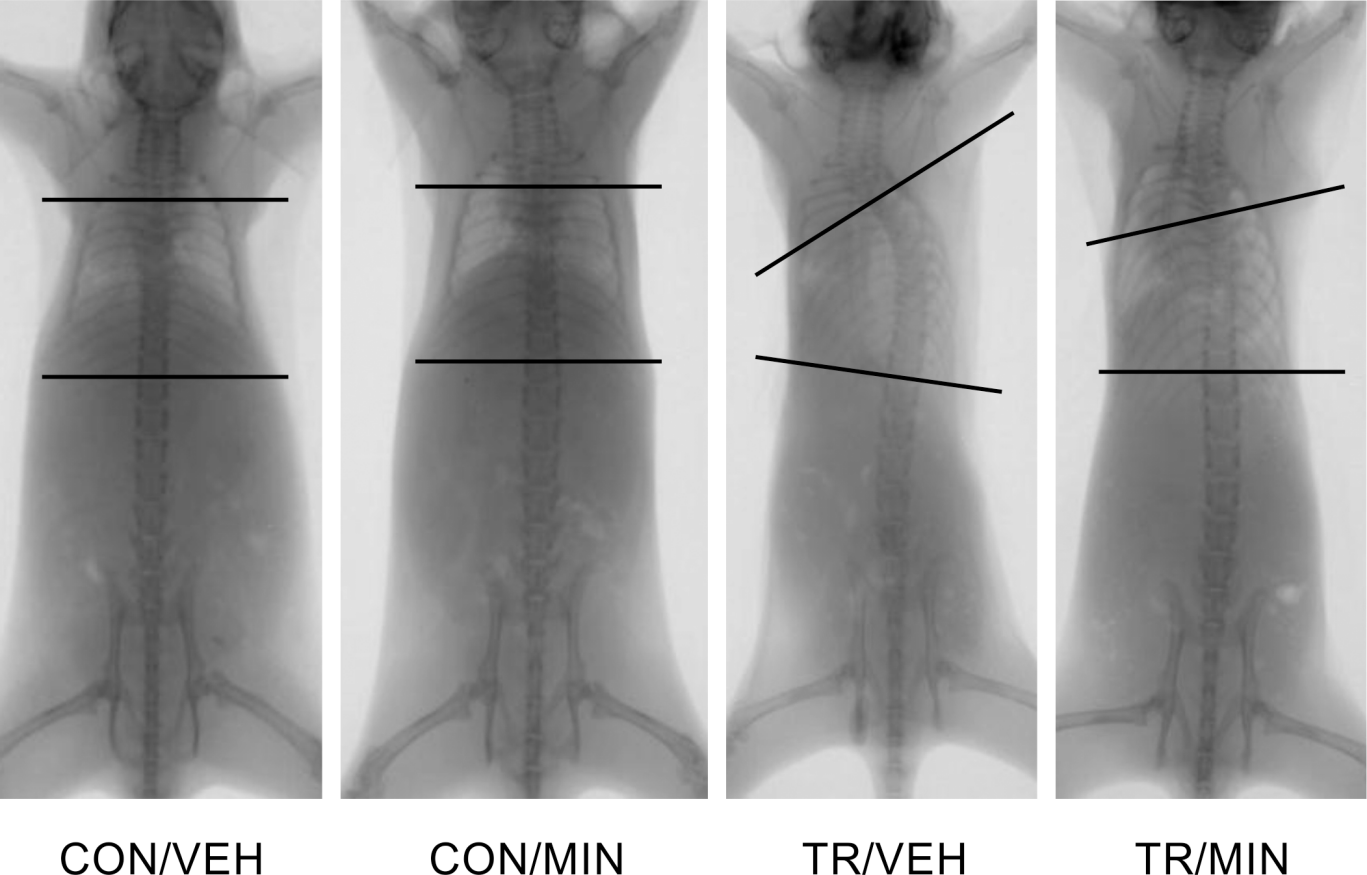
Posteroanterior radiographs of the whole spine. Cobb angle measurement of the curvatures. A straight vertebral column observed in the CON/VEH and CON/MIN mice. Scoliosis with a single thoracic curve seen in the TR/VEH and TR/MIN mice. The TR/MIN mice show lower magnitudes of spinal deformity than the TR/VEH mice.

The typical posteroanterior radiographs of the whole spine are shown in Fig 2. No mice in the CON/VEH and CON/MIN group developed scoliosis. In contrast, 28/30 (93%) mice of the TR/VEH group and 27/30 mice (90%) of the TR/MIN group developed scoliosis (Cobb angle> 10°). The apex of the scoliosis curve was consistent between the sixth and eighth thoracic vertebrae, with rotation into convexity. There was no significant difference in the incidence of the scoliotic curve between the TR/VEH and TR/MIN groups (93% vs. 90%, p> 0.999). Mean Cobb angle in the TR/MIN group was 43% lower than that in the TR/VEH group (17.9 ± 8.9° vs. 31.5 ± 13.1°, p< 0.001). Severe scoliosis (Cobb angle> 40°) was observed in 10/30 (33%) mice of the TR/VEH group, while no severe scoliosis was seen in the TR/MIN group.

### Femoral BMD in TR mice and effect of minodronate

**Fig 3:**
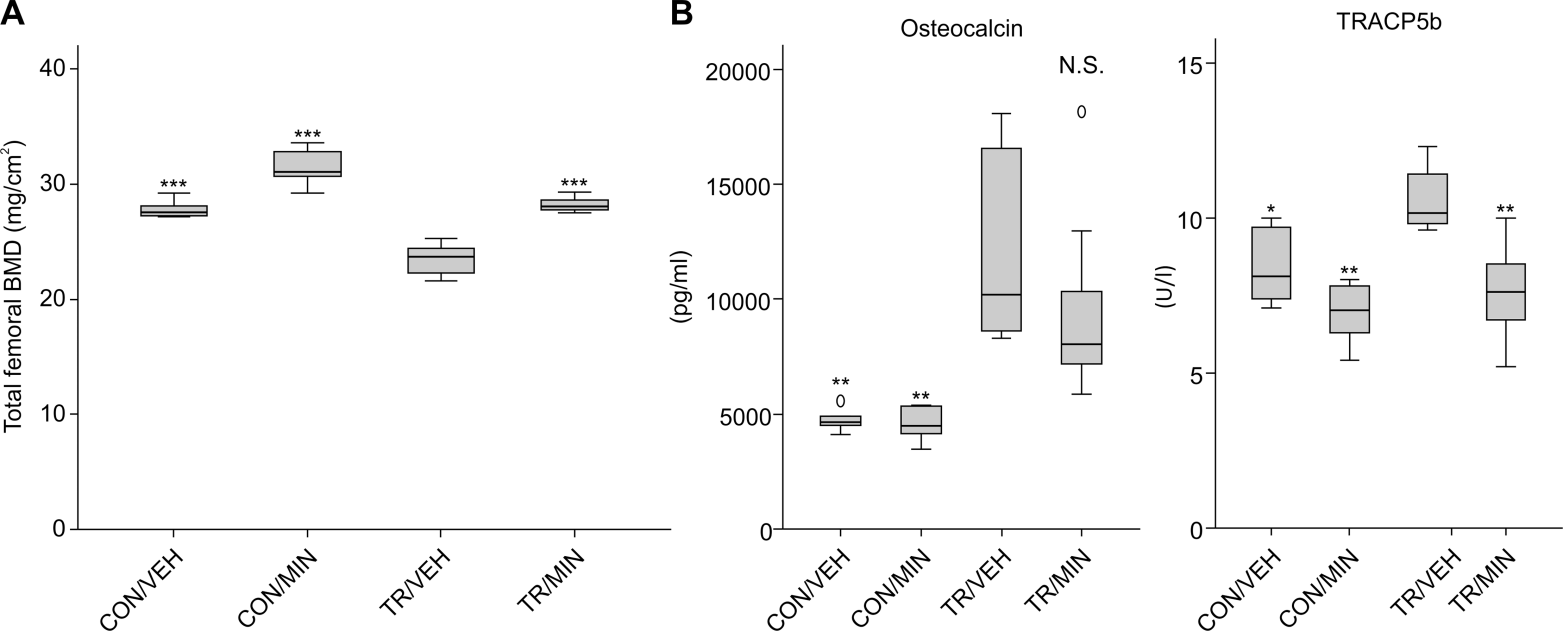
Femoral BMD in TR mice and effect of minodronate. (A) Total femoral BMD in each group at age of 9 weeks. Values shown are mean ± SD. (n = 6 for each group; ***p< 0.001, significantly different from the TR/VEH group as reference (Dunnett’s test).) (B) Effect of TR and minodronate treatment on markers of bone turnover in each group. Values shown are mean ± SD. (n = 6 for CON/VEH, CON/MIN and TR/VEH, and n = 9 for TR/MIN; *p< 0.05 and **p< 0.01, significantly different from the TR/VEH group as reference (Dunnett’s test). N.S. not significant).

Femoral BMDs in the TR/VEH group were 18% lower than those in the CON/VEH group (23.5 ± 1.4 mg/cm^2^ vs. 27.8 ± 0.7 mg/cm^2^, p< 0.001), indicating that TR induced osteoporosis in mice. In the TR/MIN group, BMD values were significantly higher than those in the TR/VEH group (28.2 ± 0.7 mg/cm^2^ vs. 23.5 ± 1.4 mg/cm^2^, p< 0.001), and were equivalent to the values in the CON/VEH group, indicating that treatment with minodronate prevented osteoporosis that would normally occur after TR (Fig 3A).

### Effect of minodronate on bone turnover markers

As compared with the CON/VEH group, the TR/VEH group had 149% higher serum osteocalcin level (11980 ± 4265 pg/mL vs. 4807 ± 668 pg/mL, p< 0.01) and 26% higher serum TRACP5b level (10.6 ± 1.1 U/L vs. 8.4 ± 1.2 U/L, p< 0.05), indicating increased both bone formation and resorption in the TR/VEH mice. Compared to the TR/VEH mice, the TR/MIN mice had 19% lower serum osetocalcin level (9644 ± 3847 pg/mL vs. 11980 ± 4265 pg/mL, p = 0.343) and 29% lower serum TRAP5b level (7.5 ± 0.5 U/L vs. 10.6 ± 0.4 U/L, p< 0.01), indicating that minodronate suppressed TR-induced accelerated bone resorption (Fig 3B).

### Bone histomorphometry in TR mice and effect of minodronate

**Fig 4:**
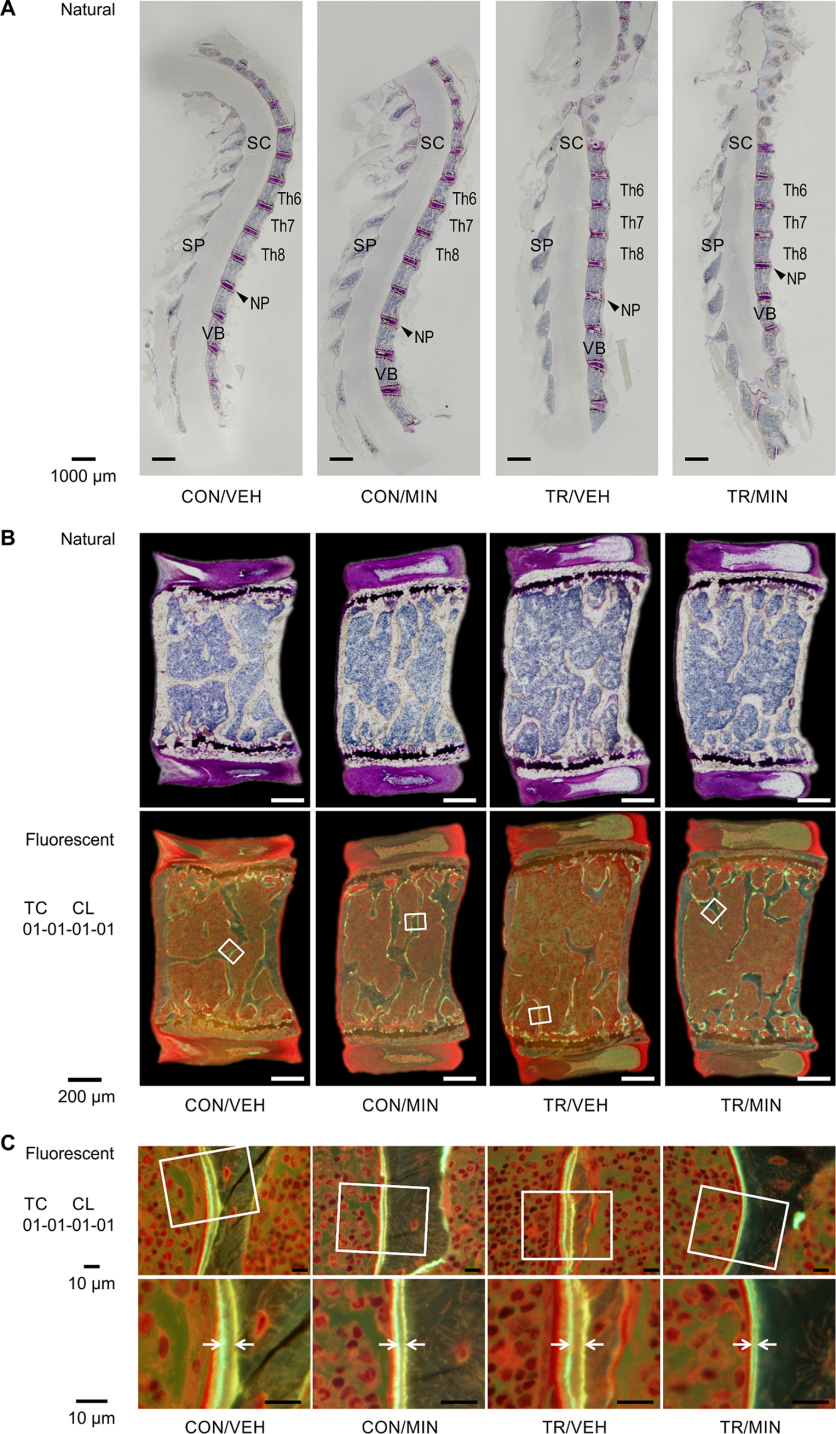
Bone histomorphometry in TR mice and effect of minodronate. (A) Villanueva staining of undecalcified sagittal sections of the thoracic vertebral column of the CON/VEH, CON/MIN, TR/VEH, and TR/MIN mice. Bar = 1000μm. NP, nucleus pulposus; SC, spinal cord; SP, spinal process; VB, vertebral bodies. (B) Histological analysis of sagittal sections of the seventh thoracic vertebral column in each group. The trabecular bone volume in the TR/VEH group is lower than that in the CON/MIN group. The decrease was sufficiently inhibited by minodronate treatment. Trabecular bone, indicated by the white squares, is highlighted in Fig 4C. Scale Bar = 200μm. (C) Fluorescent images of representative sections of the vertebral trabecular surface demonstrating double tetracycline and calcein fluorochrome labeling. The enlarged figures show the increased distance between labeled bone surfaces in the TR mice compared with the untreated control mice. Minodronate preserves TR-induced increases in bone formation. Scale Bar = 10μm.

The sagittal section of the thoracic vertebral column was obtained using Villanueva bone staining (Fig 4A). Owing to the developed lordoscoliosis, the alignment in the middle thoracic vertebral column clearly straightened in the TR groups of mice (TR/VEH and TR/MIN). Static and dynamic histomorphometry results are summarized in Table 1. TR resulted in a marked decrease in BV/TV (-43%), Tb.Th (-33%), and Tb.N (-15%) and an increase in Tb.Sp (+35%) compared with the control (CON/VEH). The decrease in trabecular bone volume was particularly noticeable at the mid-point of the vertebrae (Fig 4B.). Minodronate treatment significantly prevented TR-induced osteoporosis, as indicated by an increase in BV/TV (+113%), Tb.Th (+39%), and Tb.N (+55%), and a decrease in Tb.Sp (-46%) in the TR/MIN mice compared with the TR/VEH mice. Trabecular bone remodeling was then assessed. TR led to accelerated bone resorption, as evidenced by increased bone resorption parameters including N.Oc/BS (+38%) and Oc.S/BS (+36%) in the TR/VEH mice than in the CON/VEH mice. Treatment with minodronate suppressed the increases due to TR in all bone formation, resorption, and dynamics parameters (Table 1, Fig 4C.).

**Table 1:** Effect of thoracic restraint and minodronate treatment on static and dynamic quantitative histomorphomety of thoracic spine.

		Controls		TR	
		Vehicle	MIN	Vehicle	MIN
**Bone structure**					
	**BV/TV (%)**	20.0 ± 3.1	26.3 ± 2.7	11.5 ± 3.5 ^a^	24.5 ± 3.0 ^b^
	**Tb.Th (μm)**	38.6 ± 4.5	40.2 ± 2.0	26.0 ± 2.6	36.2 ± 2.0 ^b^
	**Tb.N (/mm)**	5.2 ± 0.6	6.5 ± 0.7	4.4 ± 0.9 ^a^	6.8 ± 0.7 ^b^
	**Tb.Sp (μm)**	156.2± 24.5	113.8 ± 14.7	210.5 ± 47.4 ^a^	112.9 ± 15.9 ^b^
**Bone formation**					
	**OS/BS (%)**	25.2 ± 6.1	13.5 ± 2.9	28.0 ± 5.5	17.3 ± 3.6 ^b^
	**Ob.S/BS (%)**	19.4 ± 6.2	7.4 ± 2.2	20.7 ± 5.1	10.5 ± 2.7 ^b^
	**O.Th (μm)**	2.44 ± 0.13	1.99 ± 0.19	2.73 ± 0.17 ^a^	1.99 ± 0.17 ^b^
**Bone resorption**					
	**ES/BS (%)**	29.2 ± 4.7	4.4 ± 2.2	34.4 ± 4.2	7.6 ± 2.9 ^b^
	**N.Oc/BS (/mm)**	3.2 ± 0.5	1.2 ± 0.3	4.4 ± 0.5 ^a^	1.2 ± 0.2 ^b^
	**Oc.S/BS (%)**	8.8 ± 1.0	2.7 ± 0.6	12.0 ± 1.7 ^a^	2.8 ± 0.4 ^b^
**Bone dynamics**					
	**MS/BS (%)**	26.3 ± 3.4	12.8 ± 3.8	30.5 ± 5.9	17.3± 2.8 ^b^
	**MAR (μm)**	1.82± 0.15	1.24 ± 0.22	2.04 ± 0.26	1.05 ± 0.22 ^b^
	**BFR/BS (μm**^**3**^**/μm**^**2**^**/year)**	174.2 ± 28.4	59.4 ± 25.0	230.0 ± 60.7	68.2 ± 22.3 ^b^

BV/TV = bone volume fraction; Tb.Th = trabecular thickness; Tb.N = trabecular number; Tb.Sp = trabecular separation; OS/BS = osteoid surface per bone surface; Ob.S/BS = osteoblast surface per bone surface; O.Th = osteoid thickness; ES/BS = eroded surface per bone surface; N.Oc/BS = osteoclast number per bone surface; Oc.S/BS = osteoclast surface per bone surface; MS/BS = mineralizing surface per bone surface; MAR = mineral apposition rate; BFR/BS = bone formation rate per bone surface; TR = thoracic restraint; MIN = minodronate

Values are mean ± SD. (n = 6 for each group; ^a^ p < 0.05 TR versus control within vehicle condition. ^b^ p < 0.05 MIN versus Vehicle within TR condition.)

### Contributing factors for the severity of the scoliotic curve

Pearson’s correlations between the Cobb angle, histomorphometric findings of the thoracic vertebrae, and femoral BMD were analyzed in the minodronate-untreated TR mice (TR/VEH group, n = 6) (Table 2). The Cobb angle was strongly, negatively correlated with bone volume (BV/TV; R = -0.82, p = 0.046) and positively with osteoclast surface (Oc.S/BS; R = 0.91, p = 0.011). The Cobb angle was also negatively correlated with trabecular thickness (Tb.Th) and femoral BMD, although these values did not reach statistical significance.

**Table 2:** Correlations between Cobb angle and contributing factors in thoracic restraint mice.

		R	p value
*Bone histomorphometry of the thoracic vertebrae*			
	BV/TV	-0.82	0.046*
	Tb.Th	-0.80	0.061
	OS/BS	0.12	0.832
	Ob.S/BS	0.03	0.964
	ES/BS	-0.15	0.783
	Oc.S/BS	0.91	0.011*
	MS/BS	0.48	0.331
	MAR	0.15	0.783
	BFR/BS	0.35	0.504
*BMD*			
	total femoral BMD	-0.72	0.115

BV/TV = bone volume fraction; Tb.Th = trabecular thickness; OS/BS = osteoid surface per bone surface; Ob. S/BS = osteoblast surface per bone surface; ES/BS = eroded surface per bone surface; Oc.S/BS = osteoclast surface per bone surface; MS/BS = mineralizing surface per bone surface; MAR = mineral apposition rate; BFR/BS = bone formation rate per bone surface; BMD = bone mineral density

n = 6

*p < 0.05

## Discussion

To our knowledge, this is the first report that investigated the effect of using bisphosphonate therapy to prevent the progression of experimental scoliotic curves in animal models. TR impaired weight gain and induced osteoporosis with increased bone resorption in mice, as evidenced by changes in body weight, femoral BMDs, serum bone marker levels, and histomorphometric findings. Treatment with minodronate led to skeletal anabolic activity in the setting of TR. The drug treatment did not affect the incidence of scoliosis (Cobb angle> 10°) but significantly reduced the curve magnitudes. In addition, severe scoliosis (Cobb angle> 40°) was not seen in the TR/MIN group. Minodronate treatment was found to be effective in preventing the progression of scoliotic curves.

Our results indicated that TR mice appeared to be a good animal model for progressive scoliosis. Kubota et al. [23] first reported that a plastic TR device was placed on the chests of 4-week-old mice, and 90% (9/10) of the mice developed progressive scoliosis at an average of about 30° at the age of 15 weeks. In pilot studies, we could confirm that TR provoked spinal curvature at 9 weeks of age with high incidence. We therefore determined that the timeframe for removing the equipment at 9 weeks of age, as performed in the present study, was the most suitable option. Additionally, as the equipment could be easily pulled off by mice, we fixed the equipment to both the dorsal and the ventral sides of the body of the mice. Although there were differences in the methods between the previous study and ours, we succeeded in confirming that TR provoked spinal curvature, with 93% (28/30) of the TR/VEH mice developing scoliosis at an average of 31.5° at the age of 9 weeks in this study.

Unexpectedly, we observed that TR caused weakened bone structures with increased bone resorption in mice. The most common methods for the induction of osteoporosis in mice are ovariectomy, tail suspension, hind limb immobilization, and glucocorticoid administration, all of which result in the loss of cancellous bone, with markedly increased bone resorption in mice [26]. Using TR in mice is an experimental method that can avoid surgery for inducing scoliosis; however, applying the equipment clearly compresses the rib cage of mice in the anteroposterior direction and reduces lung capacity, resulting in reduced weight gain and low bone mass. This is consistent with reports that chronic obstructive pulmonary disease induced by exposure to cigarette smoke causes reduced bone strength and density in growing mice [27] and rats [28]. TR use may appropriately model the airflow limitation during physical activities reported in patients with AIS with moderate or severe spinal curvature [29]. Another disease potentially related to weight reduction and low BMD is dietary insufficiency in adolescent patients, possibly caused by diseases including anorexia nervosa [30–32], which may be linked to AIS [33, 34]. Devlin et al. [35] recently reported that caloric restriction decreased body mass and impaired both cortical and trabecular bone acquisition in young growing mice. Our results suggested that TR might trigger a kind of chronic respiratory disorder and/or dietary insufficiency, reducing body weight gain and inducing osteoporosis in mice.

Many studies have investigated the effect of drugs on scoliotic curves in some animal models. A pinealectomy-induced melatonin-deficient chick model results in a spinal deformity resembling human AIS [36–38]. Aota et al. [39] proved pinealectomy induced marked cancellous bone loss with activated osteoclasts and osteoblasts in chickens. Kono et al. [38] reported that pinealectomized chickens had generalized osteoporosis compared to the controls and that melatonin restoration prevented the development of scoliosis and osteoporosis. Scoliosis can also develop in pinealectomized rats [40] or bipedal C57BL6 mice (natural melatonin knockout mice) [41]. Machida et al. [41] proved the effect of melatonin administration on reducing the scoliotic deformity occurrence rates and decreasing the rate of progression of deformity in pinealectomized models [36,40] and bipedal C57BL6 mice. SERMs including tamoxifen and raloxifene are estrogen receptor ligands that can increase bone density in humans and mice [42,43]. Previous studies showed that tamoxifen or raloxifene decreases the rate of progression of deformity in pinealectomized chickens [44] and bipedal C57BL6 mice [45,46]. Taken together, anti-osteoporosis treatment seems to be useful in controlling the scoliotic deformity in animal models.

The current study showed that the Cobb angle was strongly, negatively correlated with the bone mass (BV/TV) of the thoracic vertebrae in the TR/VEH mice. Interestingly, osteoclast surface (Oc.S/BS) of the thoracic vertebrae also strongly, positively correlated with the scoliotic deformity. The mechanism of induced scoliosis by TR has been suggested to be the imbalanced load on the vertebral body derived from the indirect mechanical effect through the ribs [23]. Asymmetric mechanical load provokes progression of scoliotic deformities according to the Hueter-Volkmann law [47]. Minodronate can suppress increases in bone turnover and improve bone strength and microarchitecture under osteoporotic disease conditions in monkeys and rats [48,49]. Our results indicated that the administration of minodronate, suppressing accelerated bone resorption and improving bone mass, might protect the vertebral body against the “imbalanced load” and finally reduce the progression of thoracic scoliosis. Although the TR mouse model is only one of several experimental animal models for human idiopathic scoliosis, this study possibly implied the clinical implications that bisphosphonate treatment could be a therapeutic option for patients with AIS.

We recently reported that 59% of human patients with AIS had higher serum level of TRAP5b than humans without AIS (> +1.88 SD), suggesting higher rates of bone resorption are common in AIS patients [9]. Additionally, in another study, we presented a detailed histomorphometric analysis of cancellous spinal process bone in AIS, and indicated that 76% of human patients with AIS had high turnover and that biomarkers of bone turnover may provide a noninvasive means for classifying AIS into specific metabolic types [50]. Taken together, we consider that bisphosphonate therapy should be preferentially adapted for treating high-turnover AIS patients before the growth of severe scoliosis is complete (i.e. Cobb angle < 20° and high values of serum TRAP5b). However, a prediction of the curve progression in high-turnover AIS patients remains poorly defined. Further studies are needed to evaluate the applicability of our findings to the clinical treatment of patients with AIS.

Some potential limitations of this study should be considered. Non-random allocation by body weight to minimize difference between the study groups at baseline may be associated with possible imbalance in baseline characteristics between the study groups. As, in a pilot study, we had noted the diversity of the severity of scoliosis induced by TR, larger sample sizes were allocated to TR-treated groups (N = 30) than the control groups (N = 20) to clarify the influence of minodronate administration on the severity of scoliosis in TR-treated mice; the different size groups may potentially cause an overestimate therapeutic effects. Additionally, this study was conducted using small sample sizes for the evaluations of femoral BMD (N = 6), serum bone marker levels (N = 6–9), and histomorphometry of the cancellous bone of the thoracic vertebrae (N = 6). To understand pathogenesis and bone metabolism in this model further, a completely randomized study, with equal sample sizes in the comparison groups and larger sample size for all of the experiments, would be needed. Future studies may also be able to assess three-dimensional bone structure in greater detail by using μCT scanning and the measurement of cortical bone of the vertebral bodies; the former would provide additional high-definition data regarding bone structure, and the latter may clarify the mechanism of the bone growth. In addition, it should be noted that the clinical characteristics of this model may not have formed a completely accurate representation of human AIS; although the severity of scoliosis was negatively correlated with bone volume of the vertebral bodies in this study, there is no evidence that AIS is always more prevalent in case of patients with poor bone status, such as those with juvenile osteoporosis. Moreover, the detailed pathomechanism underlying osteoporosis with accelerated bone resorption in this model should be elucidated in future studies.

In summary, TR induced osteoporosis with increased bone resorption in mice. The administration of minodronate increased bone mass and reduced the severity of scoliosis in the TR mice. Minodronate was suggested as a possible inhibitor of the development of scoliosis. These results may provide a novel therapeutic strategy for patients with AIS.

## Supporting information

S1 Table Experimental data set of body weights from 4 to 9 weeks of age, Cobb angle, serum bone marker levels, femoral bone mineral density (BMD), and histomorphometry of the cancellous bone of the thoracic vertebrae. (XLSX)

## References

[1] Brooks HL, Azen SP, Gerberg E, Brooks R, Chan L. Scoliosis: a prospective epidemiological study. J Bone Joint Surg Am. 1975;57: 968–972. 1194304

[2] Weinstein SL. Natural history. Spine. 1999;24: 2592–600. 10635522 10.1097/00007632-199912150-00006

[3] Rogala EJ, Drummond DS, Gurr J. Scoliosis: incidence and natural history. A prospective epidemiological study. J Bone Joint Surg Am. 1978;60: 173–176. 641080

[4] Cheng JC, Guo X. Osteopenia in adolescent idiopathic scoliosis. A primary problem or secondary to the spinal deformity? Spine. 1997;22: 1716–1721. 9259781 10.1097/00007632-199708010-00006

[5] Cheng JCY, Qin L, Cheung CS, Sher AH, Lee KM, Ng SW, et al Generalized low areal and volumetric bone mineral density in adolescent idiopathic scoliosis. J Bone Miner Res. 2000;15: 1587–1595. 10.1359/jbmr.2000.15.8.1587 10934658

[6] Cheung CS, Lee WT, Tse YK. Generalized osteopenia in adolescent idiopathic scoliosis- association with abnormal pubertal growth, bone turnover, and calcium intake? Spine. 2006;31: 330–338. 10.1097/01.brs.0000197410.92525.10 16449907

[7] Hung VW, Qin L, Cheung CS, Qin L, Cheung CS, Lam TP, et al Osteopenia: a new prognostic factor of curve progression in adolescent idiopathic scoliosis. J Bone Joint Surg Am. 2005;87: 2709–2716. 10.2106/JBJS.D.02782 16322621

[8] Lee WT, Cheung CS, Tse YK, Guo X, Qin L, Lam TP, et al Association of osteopenia with curve severity in adolescent idiopathic scoliosis: a study of 919 girls. Osteoporos Int. 2005;16: 1924–1932. 10.1007/s00198-005-1964-7 16163440

[9] Ishida K, Aota Y, Mitsugi N, Kono M, Higashi T, Kawai T, et al Relationship between bone density and bone metabolism in adolescent idiopathic scoliosis. Scoliosis. 2015;10: 1–5. 10.1186/s13013-014-0026-3 25949272 PMC4422325

[10] Weinstein SL, Dolan LA, Cheng JC, Danielsson A, Morcuende JA. Adolescent idiopathic scoliosis. Lancet. 2008;371: 1527–1537. 10.1016/S0140-6736(08)60658-3 18456103

[11] Weinstein SL, Ponseti IV. Curve progression in idiopathic scoliosis. J Bone Joint Surg Am. 1983;65: 447–455. 6833318

[12] Lu L, Dai Z, Lv G, Kang Y, Jiang Y. A novel therapeutic strategy for adolescent idiopathic scoliosis based on osteoporotic concept. Med Hypotheses. 2013;80: 773–775. 10.1016/j.mehy.2013.03.008 23562283

[13] Schiller JR, Thakur NA, Eberson CP. Brace management in adolescent idiopathic scoliosis. Clin Orthop Relat Res. 2010;468: 670–678. 10.1007/s11999-009-0884-9 19484317 PMC2816747

[14] de Mendonca RG, Sawyer JR, Kelly DM. Complications after surgical treatment of adolescent idiopathic scoliosis. Orthop Clin North Am. 2016;47: 395–403. 10.1016/j.ocl.2015.09.012 26772948

[15] Black DM, Cummings SR, Karpf DB, Cauley JA, Thompson DE, Nevitt MC, et al Randomised trial of effect of alendronate on risk of fracture in women with existing vertebral fractures. Fracture Intervention Trial Research Group. Lancet. 1996;348: 1535–1541. 8950879 10.1016/s0140-6736(96)07088-2

[16] Reginster J, Minne HW, Sorensen OH, Hooper M, Roux C, Brandi ML, et al Randomized trial of the effects of risedronate on vertebral fractures in women with established postmenopausal osteoporosis. Vertebral Efficacy with Risedronate Therapy (VERT) Study Group. Osteoporos Int. 2000;11: 83–91. 10663363 10.1007/s001980050010

[17] Matsumoto T, Hagino H, Shiraki M, Fukunaga M, Nakano T, Takaoka K, et al Effect of daily oral minodronate on vertebral fractures in Japanese postmenopausal women with established osteoporosis: a randomized placebo-controlled double-blind study. Osteoporos Int. 2009;20: 1429–1437. 10.1007/s00198-008-0816-7 19101754 PMC2708326

[18] Takeuchi M, Sakamoto S, Kawamuki K, Kurihara H, Nakahara H, Isomura Y. Studies on novel bone resorption inhibitors. II. Synthesis and pharmacological activities of fused aza-heteroarylbisphosphonate derivatives. Chem Pharm Bull (Tokyo). 1998;46: 1703–1709. 9845953 10.1248/cpb.46.1703

[19] Dunford JE, Thompson K, Coxon FP, Luckman SP, Hahn FM, Poulter CD, et al Structure-activity relationships for inhibition of farnesyl diphosphate synthase in vitro and inhibition of bone resorption in vivo by nitrogen-containing bisphosphonates. J Pharmacol Exp Ther. 2001;296: 235–242. 11160603

[20] Ohno K, Mori K, Orita M, Takeuchi M. Computational insights into binding of bisphosphates to farnesyl pyrophosphate synthase. Curr Med Chem. 2011;18: 220–233. 10.2174/092986711794088335 21110804 PMC3343387

[21] Anissipour AK, Hammerberg KW, Caudill A, Kostiuk T, Tarima S, Zhao HS, et al Behavior of scoliosis during growth in children with osteogenesis imperfecta. J Bone Joint Surg Am. 2014;96: 237–243. 10.2106/JBJS.L.01596 24500586 PMC6948836

[22] Sato A, Ouellet J, Muneta T, Glorieux FH, Rauch F. Scoliosis in osteogenesis imperfecta caused by COL1A1/COL1A2 mutations—genotype-phenotype correlations and effect of bisphosphonate treatment. Bone. 2016;86: 53–57. 10.1016/j.bone.2016.02.018 26927310

[23] Kubota K, Doi T, Murata M. Disturbance of rib cage development causes progressive thoracic scoliosis: the creation of a nonsurgical structural scoliosis model in mice. J Bone Joint Surg Am. 2013;95: e130 10.2106/JBJS.L.01381 24048561

[24] Villanueva AR, Lundin KD. A versatile new mineralized bone stain for simultaneous assessment of tetracycline and osteoid seams. Stain Technol. 1989;64: 129–138. 2480003 10.3109/10520298909106985

[25] Parfitt AM, Drezner MK, Glorieux FH, Kanis JA, Malluche H, Meunier PJ, et al Bone histomorphometry: standardization of nomenclature, symbols, and units. Report of the ASBMR Histomorphometry Nomenclature Committee. J Bone Miner Res. 1987;2: 595–610. 10.1002/jbmr.5650020617 3455637

[26] Komori T. Animal models for osteoporosis. Eur J Pharmacol. 2015;759: 287–94. 10.1016/j.ejphar.2015.03.028 25814262

[27] Akhter MP, Lund AD, Gairola CG. Bone biomechanical property deterioration due to tobacco smoke exposure. Calcif Tissue Int. 2005;77: 319–326. 10.1007/s00223-005-0072-1 16307391

[28] Ajiro Y, Tokuhashi Y, Matsuzaki H, Nakajima S, Ogawa T. Impact of passive smoking on the bones of rats. Orthopedics. 2010;33: 90–95. 10.3928/01477447-20100104-14 20192148

[29] Weinstein SL, Dolan LA, Cheng JC, Danielsson A, Morcuende JA. Adolescent idiopathic scoliosis. Lancet. 2008;371: 1527–1537. 10.1016/S0140-6736(08)60658-3 18456103

[30] Bachrach LK, Guido D, Katzman D, Litt IF, Marcus R. Decreased bone density in adolescent girls with anorexia nervosa. Pediatrics. 1990;86: 440–447. 2388792

[31] Mehler PS, Cleary BS, Gaudiani JL. Osteoporosis in anorexia nervosa. Eat Disord. 2011;19: 194–202. 10.1080/10640266.2011.551636 21360368

[32] Bialo S, Gordon CM. Effect of underweight and overweight on bone. Curr Osteopor Rep. 2014;12: 319–328. 10.1007/s11914-014-0226-z PMC5879440 24986712

[33] Smith FM, Latchford G, Hall RM, Millner PA, Dickson RA. Indications of disordered eating behavior in adolescent patients with idiopathic scoliosis. J Bone Joint Surg. 2002;84: 392–394. 10.1302/0301-620x.84b3.12619 12002499

[34] Ramirez M, Martinez-Llorens J, Sanchez JF, Bago J, Molina A, Gea J, et al Body composition in adolescent idiopathic scoliosis. Eur Spine J 2013:22: 324–329. 10.1007/s00586-012-2465-y 22886589 PMC3555626

[35] Devlin MJ, Cloutier AM, Thomas NA, Panus DA, Lotinun S, Pinz I, et al Caloric restriction leads to high marrow adiposity and low bone mass in growing mice. J Bone Miner Res. 2010;25: 2078–2088. 10.1002/jbmr.82 20229598 PMC3127399

[36] Machida M, Dubousset J, Imamura Y, Iwaya T, Yamada T, Kimura J. Role of melatonin deficiency in the development of scoliosis in pinealectomised chickens. J Bone Joint Surg Br. 1995;77: 134–138. 7822371

[37] Machida M, Miyashita Y, Murai I, Dubousset J, Yamada T, Kimura J. Role of serotonin for scoliotic deformity in pinealectomized chicken. Spine. 1997;22: 1297–1301. 9201831 10.1097/00007632-199706150-00004

[38] Kono H, Machida M, Saito M, Nishiwaki Y, Kato H, Hosogane N, et al Mechanism of osteoporosis in adolescent idiopathic scoliosis: experimental scoliosis in pinealectomized chickens. J Pineal Res. 2011;51: 387–393. 10.1111/j.1600-079X.2011.00901.x 21649717

[39] Aota Y, Terayama H, Saito T, Itoh M. Pinealectomy in a broiler chicken model impairs endochondral ossification and induces rapid cancellous bone loss. Spine J. 2013;13: 1607–1616. 10.1016/j.spinee.2013.05.017 23791240

[40] Machida M, Murai I, Miyashita Y, Dubousset J, Yamada T, Kimura J. Pathogenesis of idiopathic scoliosis. Experimental study in rats. Spine. 1999;24: 1985–1989. 10528372 10.1097/00007632-199910010-00004

[41] Machida M, Dubousset J, Yamada T, Kimura J, Saito M, Shiraishi T, et al Experimental scoliosis in melatonin-deficient C57BL/6J mice without pinealectomy. J Pineal Res. 2006;41: 1–7. 10.1111/j.1600-079X.2005.00312.x 16842534

[42] Delmas PD, Bjarnason NH, Mitlak BH, Ravoux AC, Shah AC, Huster WJ, et al Effects of raloxifene on bone mineral density, serum cholesterol concentrations, and uterine endometrium in postmenopausal women. N Engl J Med. 1997;337: 1641–1647. 10.1056/NEJM199712043372301 9385122

[43] Starnes LM, Downey CM, Boyd SK, Jirik FR. Increased bone mass in male and female mice following tamoxifen administration. Genesis. 2007;45: 229–235. 10.1002/dvg.20294 17417806

[44] Akel I, Kocak O, Bozkurt G, Alanay A, Marcucio R, Acaroglu E. The effect of calmodulin antagonists on experimental scoliosis: a pinealectomized chicken model. Spine. 2009;34: 533–538. 10.1097/BRS.0b013e31818be0b1 19282733

[45] Akel I, Demirkiran G, Alanay A, Karahan S, Marcucio R, Acaroglu E. The effect of calmodulin antagonists on scoliosis: bipedal C57BL/6 mice model. Eur Spine J. 2009;18: 499–505. 10.1007/s00586-009-0912-1 19242737 PMC2899472

[46] Demirkiran G, Dede O, Yalcin N, Akel I, Marcucio R, Acaroglu E. Selective estrogen receptor modulation prevents scoliotic curve progression: radiologic and histomorphometric study on a bipedal C57Bl6 mice model. Eur Spine J. 2014;23: 455–462. 10.1007/s00586-013-3072-2 24136418 PMC3906449

[47] Mente PL, Stokes IA, Spence H, Aronsson DD. Progression of vertebral wedging in an asymmetrically loaded rat tail model. Spine. 1997;22: 1292–1296. 9201830 10.1097/00007632-199706150-00003

[48] Mori H, Tanaka M, Kayasuga R, Masuda T, Ochi Y, Yamada H, et al Minodronic acid (ONO-5920/YM529) prevents decrease in bone mineral density and bone strength, and improves bone microarchitecture in ovariectomized cynomolgus monkeys. Bone. 2008;43: 840–848. 10.1016/j.bone.2008.07.242 18718565

[49] Kimoto A, Tanaka M, Nozaki K, Mori M, Fukushima S, Mori H, et al Intermittent minodronic acid treatment with sufficient bone resorption inhibition prevents reduction in bone mass and strength in ovariectomized rats with established osteopenia comparable with daily treatment. Bone. 2013;55: 189–197. 10.1016/j.bone.2013.02.013 23486179

[50] Tanabe H, Aota Y, Nakamura N, Saito T. A histomorphometric study of the cancellous spinal process bone in adolescent idiopathic scoliosis. Eur Spine J. 2017:26: 1600–1609. 10.1007/s00586-017-4974-1 28150052

